# Corrigendum

**DOI:** 10.3402/gha.v8.29398

**Published:** 2015-08-25

Regarding the paper titled: “‘You sit in fear’: understanding perceptions of nodding syndrome in post-conflict northern Uganda” by Kristine Buchmann

Published in *Global Health Action (*section “Original Articles”) 21 October 2014. Citation: Glob Health Action 2014, **7**: 25069 - http://dx.doi.org/10.3402/gha.v7.25069

In the third paragraph, the reference at the end of the third sentence is incorrect and the correct reference is missing from the list of references.

This sentence currently reads:

Onchocerciasis has previously been associated with seizures and stunting; a recent meta-analysis showed that for every 10% increase in the prevalence of onchocerciasis, epilepsy rates go up by 0.4% (8).

The section should read:

Onchocerciasis has previously been associated with seizures and stunting; a recent meta-analysis showed that for every 10% increase in the prevalence of onchocerciasis, epilepsy rates go up by 0.4% (13).

Note: this change results in a renumbering of the citations throughout the rest of the article. The full article with the correctly numbered citations is provided below.
