# Supplementary material for: Corrigendum
Source: Glob Health Action. 2015 Aug 25;8:10.3402/gha.v8.29398. doi: 10.3402/gha.v8.29398 (PMC4550674; doi:10.3402/gha.v8.29398)
Supplement: Related texts

# Sub article: ‘You sit in fear’: understanding perceptions of nodding syndrome in post-conflict northern Uganda

**DOI:** 10.3402/gha.v7.25069

**Published:** 2014-10-21

**Authors:** Kristine Buchmann

**Keywords:** nodding syndrome, onchocerciasis, seizure, perceptions, emerging disease

## Abstract

**Background:**

Nodding syndrome, a disabling epidemic epileptic encephalopathy, has affected an estimated 1,834 children in northern Uganda, with reports of as many as 3,000. Etiology is unknown and children are being treated symptomatically but inconsistently with anti-epileptic drugs.

**Design:**

This qualitative study comprised 10 semi-structured interviews with caregivers of affected children and five focus group discussions with 23 participants; relatives, teachers, and religious leaders. Data collection and participant observation were carried out from July to September 2012 in Kitgum and Pader districts. The material was coded through inductive thematic analysis.

**Results:**

Nodding syndrome has brought signs of discrimination in school admission procedures, founded in a fear of transmission. The suffering and loss caused by nodding syndrome is collective, and participants felt that nodding syndrome was viewed as a threat to the Acholi only, and that interventions had therefore been delayed. Multiple theories of causation exist, most commonly that the disease is caused by chemicals from bombs or that food aid distributed in IDP camps had expired or been poisoned.

A feeling of uncertainty was present in all focus group discussions, fueled by the fact that results of investigations were not being shared with the communities. It was especially agonizing that CDC results had been given to the Ugandan government in 2010 but not to the public. The definitive fear is that the disease will be the end of the Acholi.

**Conclusions:**

This study provided insight into the perceptions of communities affected by an unknown emerging disease. Families of affected children are grieving not only their child's illness; it is a loss of social value and of lineage. The loss and suffering involved with nodding syndrome should be seen in the context of the wider suffering of a society disrupted by violent conflict. The memory of war is omnipresent and is also how nodding syndrome is understood.

Nodding syndrome is a neurological syndrome of unknown pathogenesis and prevalence, devastating communities in Eastern Africa. It predominantly affects children aged between 5 and 15 and causes stunting and mental retardation (1). Abnormal brain activity results in a brief lapse in neck muscle tone, causing the head to nod forwards. These seizures seem to have been activated by the sight of food, and therefore, the children shy away from meals and suffer from malnutrition. Many develop generalized seizures and thus are prone to accidents such as falling into fires or rivers, which has caused many deaths. There is no cure and once a child is affected he or she gradually loses interest in the surroundings, while some develop progressive encephalopathy and lose cognitive functions, permanently changing the child's behavior (1, 2).

By March 2013, it was estimated that at least 1,834 children in northern Uganda were living with nodding syndrome (3). Local health staff report up to 3,000 cases and that an estimated 200 have died (3–5). It was first described in southern Tanzania as early as 1962, briefly in 1983 in Liberia, and in south Sudan since 1997 (2). The clinical studies that have been carried out have shown cerebral atrophy, gliotic lesions, and occasionally, hippocampus sclerosis; the syndrome is now called an epidemic epileptic encephalopathy (1, 2, 6, 7).

The main theory is that the syndrome is associated with the parasite *Onchocerca volvulus*, which is spread by the black fly and causes river blindness. A large proportion of children and adults in the affected areas are infested with this parasite, but studies have failed to find significantly elevated antibodies to the parasite in the cerebrospinal fluid of affected children (1, 2, 4, 6, 8–12). Onchocerciasis has previously been associated with seizures and stunting; a recent meta-analysis showed that for every 10% increase in the prevalence of onchocerciasis, epilepsy rates go up by 0.4% (13). Onchocerciasis is one of the most commonly neglected tropical diseases, and it infects 37 million people worldwide (14). It is not clear why people living in other endemic regions have not experienced outbreaks of nodding syndrome, or why it is occurring in the affected regions at this moment in time (4, 15). People have speculated, but no evidence has been found, that the syndrome could be caused by viral encephalitis, chemicals from the heavy artillery used in the wars in southern Sudan and northern Uganda, the food aid that was distributed, vaccines, eating bush meat, or using unclean water (4, 15). It is also being investigated whether vitamin B6 deficiency, which is common among the children, could be linked (15).

In northern Uganda, the symptomatic treatment of nodding syndrome with anti-epileptic drugs began in March 2012 when a treatment center was opened in each of the five affected districts, after intense pressure by politicians from the North. Outreach clinics ran from June 2012 to February 2013 when money for fuel was halted by the government.

Three qualitative studies and a commentary have been published; all after the present study began. The first used checklists and questionnaires to examine the views of health workers and parents in focus group discussions. The method of analysis is not clear. It also mentions the experience of children suffering from nodding syndrome, but does not say how many were interviewed and how. The majority of health workers believed that nodding syndrome was caused by psychological trauma from the war, undernutrition during camp life, or the black fly (16). The commentary is a reflection on quotes collected through focus groups – it is not clear which method of analysis was used. The participants reportedly felt that food aid or chemicals from bombs caused nodding syndrome, but also mentioned spiritual and religious beliefs (17). An anthropological study looked at processes of labeling and defining nodding syndrome. They found that in the academic literature on the topic, a biomedical discourse and language use is prominent, but that in the local explanatory models nodding syndrome is being linked to social issues. They state that nodding syndrome has come to symbolize the marginalization of a region and has become a political tool (18).

From 1986 to 2008, the northern region of Uganda was affected by the atrocities of the anti-government Lord's Resistance Army. During the conflict, family and societal structures were altered and up to 30,000 children were abducted by rebels and used as soldiers and sex slaves (19). Approximately 2 million people were forcibly displaced by the government into overcrowded camps, resulting in 1,000 excess deaths per week in 2005, the majority caused by curable diseases and malnutrition (20, 21). The majority of people left the IDP camps in 2009 and moved back to their villages to start their daily lives again. The government has been accused of human right abuses and of failing to protect the people living in the IDP camps (20, 22–24). North–South divisions have existed since independence in 1962 (23, 24), and some politicians from the North have recently started a petition for the North to be separated as a new country (25).

In Uganda, nodding syndrome was first found in the districts of Pader and Kitgum and that is where the majority of the affected children are still to be found, and therefore, where this study was carried out. Nodding syndrome has since been confirmed in three other districts in northern Uganda.

## Methods

Data triangulation was performed in order to increase the chance of achieving thick descriptions. The researcher started with an unrecorded key informant interview with an Acholi chief, which helped guide her on the contemporary beliefs surrounding nodding syndrome, and confirm the decision not to interview traditional healers, as they were no longer very involved in the treatment of the affected children. Several gatekeepers were encountered, and health workers had been banned from giving information to journalists. Using purposive sampling, participants were identified in treatment centers and during outreach clinics. Through the snowball method participants were asked to identify cases from their own social networks.

Ten qualitative semi-structured interviews and five focus group discussions were carried out in Kitgum and Pader districts between July and September 2012. In total, 33 people participated and 9 extra people became key informants with whom the researcher kept in touch. All data collection was carried out in English (the official language in Uganda).

Participants in the semi-structured interviews were primary caregivers of children aged between 5 and 17 with nodding syndrome (three fathers, three mothers, an aunt, two older brothers, and an older sister, all from different families). The interview guide focused on how it had affected the child, siblings and parents, the child's opportunities of participating in social activities, reactions received from neighbors, and their understanding of causation.

The focus groups consisted of two groups of relatives, two groups of primary school teachers, and one group of religious leaders, each with 4–6 participants. The religious leaders represented the following denominations: Anglican, Catholicism, Pentecostal, Seventh Day Adventist, and Islam. The thematic interview guide focused on their understanding of the disease, why they think it has occurred to the children, and how it has affected their communities. The individual interviews lasted an average of 101 min and the focus groups 122 min.

Saturation was reached at a fairly early point, but data collection continued to ensure that findings in the next district would not be different. To add to the data trail, field observations were collected as the researcher did participant observation with the MoH outreach clinics that had just begun. The recorded interviews were transcribed verbatim by the researcher in preparation for analysis. This generated 263 pages of single-space data plus 5 pages of notes from field observations, which were analyzed using inductive thematic analysis. This method was chosen in order to give an accurate reflection of the content where themes are not shaped by the researcher's preconception or by a theoretical framework (26).

A large number of codes were derived from the material and nine themes were developed from the inductive analysis described earlier. They were condensed into six themes, only omitting the theme called ‘Changes brought by war’ because of space limitations.

Permission for this study was obtained from Gulu University's Institutional Review Board and the Ugandan National Council for Science and Technology. Participants were given oral and written information describing the study, and written consent was obtained from all participants. To preserve confidentiality of the informants, the specific villages remain anonymous. A transport refund was provided to all participants. A parallel article entitled ‘These nodding people: experiences of having a child with nodding syndrome in post-conflict northern Uganda’ stems from the same study.

## Results

The citations came from both Kitgum and Pader districts and no clear difference between people's perceptions was found.

### Helplessness

#### Uncertainty

The many expressions of uncertainty show how people in all layers of society are trying to make sense of this predicament that has such a huge influence on their society.

Now … people can go up to the moon and back. And if the person is seriously sick (…) they can operate and take out that sickness and the person survives. In the case of this nodding disease, as there are very many physicians in the WHO, if they come here can they not find out the cause of this nodding disease? (Religious leader, Seventh Day Adventist)

Results of investigations are not being disseminated, not even to the people whose children were tested, and participants portrayed a low comprehensibility. A father recounted that also the local hospital did not give feedback on postmortems.

In an attempt to bring attention to the thousands of children suffering from nodding syndrome who had not yet started receiving treatment, an MP took initiative to transport 22 affected children to the national referral hospital in Kampala in March 2012. Police stopped them twice on their way, making them wait in the vehicle for hours for permission to proceed. Because the parents were given no explanation for this detainment, they felt that their problems were not allowed to be exposed. Some of the children eventually formed the basis of the study by Idro et al. (6).

It is also irritating people politically because there is no proper government intervention. The data, you go to the district here and you ask for the data, nobody will avail you with the exact data of what is on the ground (…) That makes people sceptical, why, why the secret? This thing should be the concern of every population of this country. (Religious leader, Pentecostal)

The majority of participants expressed a feeling of being kept in the dark, and many recounted how the aforementioned MP had revealed that the results of the 2009 CDC investigations had been given to the Ugandan government in 2010, but were not being shared with the public – a fact that was also stated in Ugandan media (27). The results were published in March 2013.

We as Acholi we have protested this government, that's why people are thinking might be the government has done something … because the specimen which was taken to Atlanta for more investigation, the result is not given back, why?! And our Woman MP went right up to Atlanta, from there they said they have already released the result, the result is here now in Uganda, but the government does not want to release this result. (Father of 17 yr. old CWNS)^1^

In August 2012 when interviews for this study were taking place, a conference on nodding syndrome was held in Kampala, results were shared and representatives from the Acholi group were present.

#### Lack of help

Several participants stated that the Acholi are being marginalized by the government because of old power battles. Some said that a new dangerous weed (*obuga*) had been brought into the Northern region, deliberately mixed with seeds distributed during the war, which now made it impossible for anything else to grow. Other participants said it had always been around. At the time of the interviews, a small Ebola outbreak occurred in western Uganda, claiming 17 lives and receiving immediate national and international media attention. A brother of an affected child suggested that because the North gave the ruling government more votes, now in an attempt to be included, it deserved to be helped.

Like for Ebola it has broken out in Kiibale and just within a few days they have released billions of shillings, around 600 billion! To help all these health workers there and the people who are having the problem. But *here*, it took *long* period for Northern region to receive support from central government. So there is that gap (…) That is why we tried to open their eyes by saying that this issue of nodding disease could be raised, because last time we participated highly in voting.

Several statements of how nodding syndrome had become a ‘political disease’ appeared in the material. Reflecting on the recent corruption scandal in the prime minister's office and the fact that food support for the patients had stopped, one participant said: ‘You will find they are eating public funds, which should be used to help these local people’ (Brother of 1 CWNS).

### Theories of causation and transmission

Key informants explained how traditionally in Africa it is not ‘what’ has made me sick, but ‘who’. Disease is not thought to strike randomly, but rather as a consequence of a disruption in the social or moral order, which must be repaired.

During the insurgency in northern Uganda, large numbers of people were killed in brutal ways. It is believed that when someone dies a violent death or does not receive a decent burial, his spirit (*lacen*) will linger and may enter another person. When the 2 million people who were living in IDP camps returned to their homes after the war, they came across bones and are very aware that they are living near spirits of the dead. In the following quote, an older brother describes the day his sister first started nodding.

At around midday she started shouting saying that she is seeing an old woman who is burning in a fire, in a very hot fire and running away, but for us we are not seeing the thing (…) So there is bad spirit actually. There is a linkage between this war and the disease. And after saying that she has seen a woman in hot fire she started nodding. (Brother of 2 CWNS)

Misfortune is sometimes believed to be caused by immoral acts that an individual or his ancestor has committed in the past. Everyone in the focus group of relatives in Kitgum district had a relative who had been abducted and forced to fight for the rebels, but they said that those acts could not be the ones that were making the children sick now. Some suggested that nodding syndrome might be a punishment from the ancestors because their shrines were destroyed during the war. Similar to other feelings of being marginalized some expressed a fear that people with bad intentions in other regions or countries might have sent a curse to do away with the Acholi. The belief that disease never strikes randomly coupled with a long-term feeling of marginalization and a very external locus of control are clues to the context of the high number of theories. All participants said that nodding syndrome has come as a result of the war.

They said it was in West Africa in Sierra Leone, then in Sudan also, then another one it was where …? A certain country they were talking on the radio. But those were also the countries that have suffered war. That is why we said why? Why is it that the nodding disease affects people after the war, why after the war huh? (Father of 13 yr. old CWNS)

The quote from the following mother shows the pluralistic attempts of meaning-making; she did not believe that chemicals could have affected her while pregnant, instead she believed that her son's nodding syndrome was caused by sleeping outside without protection from insects during the war when he was a baby, and by the spirits of those who died a violent death:

At night-time we always sleep in the bush, 2000–2001–2002 we were in the bush (…) Only one of my children are affected that is why I can't think that it affected [him] in my stomach. Maybe it affected from outside. For me I think from the demons. Because you know some places from our side, they killed many people at *once*, even 30 peoples, you will find that they tie them and put them in the house, burned them – so maybe the demons? (Mother of 13 yr. old CWNS)

The chiefs in the region have carried out traditional rituals in an attempt to appease the evil spirits. None of the participants believed that further rituals or herbal medicine would make a difference because it was tried in vain at the beginning of the epidemic. The aforementioned mother believed that the way forward is medicine and her Christian faith:

For me I just only put on prayer, because everything come from God (…) Some people who are witchdoctors their children are also affected with this thing. So where can we go to? Only God that we can look to.

Informants reported that the illness was first noticed in the IDP camps, and some linked the cramped conditions, malnutrition, rubbish leaking into wells, etc., to its occurrence. ‘And very many people were killed and were not buried along the river, they just fall in that river, and then people are using that water even for consumption’ (Religious leader, Islam). Also in this view, nodding syndrome is understood through understanding the consequences of war. A few mentioned the possibility that it could have been brought by people who traveled to and from south Sudan. The theory mentioned by almost all participants was that the weapons used by the government during the war had left chemicals in the air, water, or plants which then affected pregnant women and/or children.

Because normally, after firing, the smoke can go up and then it rains, normally rain water brings down the smoke and when they are brought down they can flow to the river, to the stream, in the well and they get accumulated there (…) Ok in the air it can be by breathing. And through eating the fruit from the plant. And maybe by drinking water. (Teacher C)

In line with the plural health beliefs most participants inhabited at least two theories of causation simultaneously. The second commonest theory was that food aid distributed in the IDP camps was expired or poisoned. The maize flour had been bitter or smelt odd and often the sacks had no expiry date on them, or they did not trust that it had not been tampered with after leaving the supplier.

Something good for someone to eat, there must be a date manufactured and expiry date. It must be there. And this one being given, there is no expiring date and manufacturing date, there is no, nothing there. (Brother to 1 CWNS)

All informants said this was not a fear at the time when they had no choice but to consume it, but is something that has come to their minds now as they search for answers. They however described vividly how they had been puzzled by certain findings.

Although participants had lived in different IDP camps, they had all heard that broken bottles had been found in maize flour in one camp, and this was seen as a clear sign that there were deliberate attempts to harm them.

You know there is a lot of suspicion. People are thinking maybe this is something done silently to finish the population. Because people were getting literally things like broken bottles, even what, things that people could not even explain – in the food (…) So people are thinking, maybe because the resistance to this government was based here, maybe this was, you know, the way they wanted to pay us back, with you know, this silent war now. (Religious leader, Pentecostal)

Both in the IDP camps and now during nodding syndrome food distribution, small sachets of chemicals were found inside the sacks of beans. The community raised their concern but was told the sachets contained pest control. Mistrust was expressed again in the quote ‘But for them they say that powder there is for food preservation [laughter]’ (Cousin and brother to 4 CWNS).

In this region, they use sesame oil for cooking, thus liquid sunflower oil already seemed to be of less nutritional value to them and when tins of solid oil were distributed it brought great anxiety.

Yeah the cooking oil they are in liquid shape, but the other one you can see they are compact together, they are in solid I can say. So when you put it on the sun it can melt and go back to their normal shape. And the smell actually is different. It is written on the tin ‘Palmolin’, I don't know even that tree, is it palm tree or what I don't know. (Brother to 2 CWNS)

This quote implies that distributing solid Palmolin and maize oil that are unknown in the area without informing people of its origin has proven to be a breeding ground for suspicion years later. Helplessness was expressed throughout the interviews:

You would ask yourself ‘Why is it that mine is like fat when the other person's is liquid?’ Thereby it comes into your idea that maybe this cooking oil has what, been contaminated. But you could not do anything. You have to eat so that tomorrow you continue. (Teacher D)

Participants were open to the notion of the black fly as a cause of nodding syndrome, especially those who had gone through health education. But they did not generally believe the black fly to be the cause, reasoning that then nodding syndrome should have affected them years ago, or adults now too. Many however made the observation that along rivers there are high numbers of affected children, coherent with the fact that black flies breed in highly oxygenated water.

### Changes brought by the disease

#### Changes to education

Nodding syndrome has affected the education system in several ways. One of the complexities of nodding syndrome is that seizures are sparked by both the sight of food and by hunger. Teachers in one focus group illuminated a structural barrier: because many children do not eat breakfast and lunch is not served in school, from 11 am and onwards a child starts nodding in every class. Teachers said they had advised parents to pack cassava snacks for their children, but that parents are unable to do so because of poverty. Most affected children end up dropping out of school, but also healthy children are kept at home due to fear of infection. Teachers struggled to agree on whether the affected children should be isolated for infection control and to reduce the daily interruptions.

If they are attacked, the whole day in school they are affected and (…) in fact the whole class is disorganized, the mind of the children are distorted, so to bring the mind of the children back to the lesson is very hard. So it means it affects the whole pupils in the school, in the class, so they should have to be isolated until the condition is calm. (Teacher I)

When asked if they would take the job as teacher for isolated children, they all agreed that would be a stigmatized position to hold. When asked if a salary increment would make a difference, they said they would first have to consider if the savings from that increment would be enough to help their families after they had died in case they got affected. Being a teacher for children with HIV was said not to be stigmatized because the mode of transmission is known, but being a teacher for mentally ill was.

And if it is possible these children should be given a special school (…) if they are included in a school setting like this one, the teacher may even be nicknamed as ‘Nodding teacher’ and the children as ‘Nodding pupils’. (Teacher D)

This conversation in an FGD with teachers in Kitgum district showed that the fear of nodding syndrome has brought changes to school admission procedures, with signs of discrimination both in their district and in the capital Kampala:

If the child is affected it will not be admitted. I think it only remains the village schools where they admit everyone, but in town schools now they are selecting (Teacher H). And it has gone up to the national level, schools in Kampala. They are not admitting children from Kitgum who are suffering from nodding disease (Teacher G). When you are joining schools these days you must have the medical form now filled from the doctor. Because in the past we didn't have that in primary schools. Actually I don't know; by next year it may come up to nursery schools. (Teacher H)

#### Fear

A key informant recorded 124 children with nodding syndrome out of 972 inhabitants in Tumangu village, Kitgum district. No one in the community is free from worrying about this disease. The biggest threat to the community therefore may not be sick children; but a stigma that further erodes the fabric of their culture, a reduction in farming that will affect the future of entire families, and the constant stress of an unknown threat:

That means the social or economic activities have gone down because of that disease. Actually the set up, the whole set up of the people have changed. This is because now *you sit in fear* – maybe tomorrow it will be my son or it will be my child. So every time I am tense, you fear. (Teacher H)

The statement below shows that trauma is still occurring on a daily basis, no longer due to insecurity but due to the sights and stories that people are faced with.

And every time, people in the community are seeing horrible sights. Because there are girls I saw with my eyes, who were burnt, they lost part of their eyes, their breast, even their hands – not there! It's unspeakable. So when you see this on a daily basis, you who is normal, you also get the secondary trauma. (Religious leader, Pentecostal)

#### Neglect

During data collection many examples of neglect and abuse appeared. The following incident in Pader came out during one of the interviews and was confirmed through a home visit. It had not been reported to the outreach team or police, contrary to what happens if a healthy child suddenly goes missing.

And some other children died in the forest. Like the one in July. Their parent told him ‘you go back, you go and stay at home’. But he is now tired and he falls near home (…) For two weeks people were looking for him but they don't find. One day, they saw the dog coming, when the dog eat too much. They said ‘Eh, *what* has that dog eaten?’ And they followed that dog another day, and they find remaining the head of that child. (Mother of 13 yr. old CWNS)

Most likely the child had died from hypoglycemia after losing his way in the tall grass during postictal confusion, just 500 meters from home. The researcher also saw cases of abandoned children who were found incontinent and unable to speak after being left alone in a hut. Parents are strained to their limits and a mentally disabled child was often described as being of no value.

And another thing from here, the moment they realize that this child is sick, this nodding disease, it is very difficult. There will be segregation, saying that child is useless, it's a dead person moving, there is such kind of thinking. (Aunt to 9 yr. old CWNS)

The words that were used when the researcher talked to people about the syndrome were as harsh as the condition.

Noted many times that people use these words when describing the children: ‘useless’ ‘wasted’ ‘like dog’ 'wild’ ‘rude’ ‘hopeless’. (Field note, Kitgum, 24.9.12)

Several affected young girls who were not alert during absence seizures had become pregnant as a result of rape, some seen bringing their infants when collecting medication. As the affected boys reached puberty some started sexually assaulting others, and some were taken to prison.

### A lost future

The definitive fear that this is a lost future is described in this theme.

And for us in our family there is 7. Out of 10 we have only 3 children that are okay. The 7 are useless as I see, the future of them to educate now, because they don't have memory. (Uncle to 7 CWNS)

The worry was expressed as a concern for the community, the children in general, and the tribe. This emphasis on the group rather than on the individual could strengthen a community who has to go through something like this, but it might be harder to be a disabled child in one. Children represent a great value in being the adults’ best hope for a secure future. A severely impaired child therefore represents lost potential, a current drain on resources as well as a future burden. Several requests for assistance to the communities and help in retrieving the CDC results were given to the researcher.

But the only thing which I need to add; when you go back don't forget about northern Uganda, as you have seen that people are traumatized. Don't forget. You need to come and help us; otherwise we are going to die. Because after the children has died, we the parents we are useless. (Uncle to 7 CWNS)

The feeling of helplessness is echoed in this theme; the affected children are useless, parents are useless without children, we are all hopeless. The suffering is also collective – even the stigma is shared in the sense that only the Acholi group is affected. A region that was finding its feet after two decades of war is now facing another threat, expressed in the following quote that contains words often used to describe war or weapons of mass destruction:

People are now seeing a lot of danger that there will be no continuation of their family lineage. Because if all the children are now affected, this generation of our children will be wiped out. (Religious leader, Pentecostal)

The following father had 15 children and 6 of them had died from meningitis and malaria. He expressed the definitive fear that this disease could be the end of the Acholi people:

Well our main worry in fact if this disease cannot be cured, then automatically our future is not there, there will be no children (…) so maybe in some 10 or 20 years there will be nobody around here … Because it is only we the old people [laughter] who are not affected, so how long do you think we can stay [alive]? We cannot stay. Therefore if there is no cure and they all die then automatically there will be nobody here. (Father of 13 yr. old CWNS)

## Discussion

### Discussion of method

The researcher acquired an intimate knowledge of the material by carrying out the data collection, transcription, and coding herself. Had the interviews been done in the local language Luo, people with no primary education at all could have been included which might have meant that the notion of spirits would have been more dominant. Besides being peasant farmers, five participants were also unpaid volunteer health team members (people with no medical education who are asked to distribute medicine) and had attended an information meeting about nodding syndrome.

### Discussion of results

Participants felt that their sense of uncertainty and helplessness was increased by the fact that results from clinical studies carried out on children in their region were not being disseminated. They also believed that the government and international society viewed nodding syndrome as a threat to the Acholi only, and that interventions were therefore being delayed. Participants lamented the fact that this has become a political disease, and many explained how political slur stood in the way of interventions. Local politicians from opposing parties had found power struggles in who was allowed to make which decisions or say what to the media.

The theories of causation listed in this study resemble findings in the four qualitative studies published (16–18, 28). The strong fear that the food distributed was expired or poisoned indicates that if unknown types of food are distributed in an area it must be accompanied with information and clearly marked with expiry dates. The feeling of being targeted is important to be aware of, because in an environment of distrust people might also not trust well-intentioned interventions such as mass distribution of the drug against onchocerciasis. Almost all participants listed chemicals from bombs, or food aid that had been purposely poisoned, as their theory of causation – also in this framework war is how nodding syndrome was understood. This is similar to findings by Bukuluki et al. (28). In the framework of disease being caused by social disruptions, nodding syndrome looks like the younger generation's ‘inheritance’ of disturbances caused by the war, and in the same way the disease could burden generations to come with the spirits of those who now die the nodding death. Participants in this study understood biomedical aspects of epilepsy and other diseases, but felt it did not explain everything. A recent study on mental health found that 8.2% of a general population in northern Uganda reported high levels of spiritual possession (29). This means that in that area, feeling possessed by a spirit was as common as being infected with HIV, thus believing in spirit possession might be a highly prevalent and underestimated condition in post-conflict areas. It has been argued that spiritual propaganda by the Lord's Resistance Army has contributed to the fear that spirits may be ever present (29).

In the themes called ‘Theories of causation and transmission’ and ‘Changes brought by the syndrome’, it was shown that there is both a fear of transmission and a stigma associated with nodding syndrome. The education of affected children as well as classmates is disrupted by the commotion caused when a child has a nodding spell or seizure, similar to findings by Bukuluki et al. (28). The present study additionally found that the emergence of nodding syndrome has brought discrimination to school admission procedures because of a fear of transmission.

The physical manifestations of nodding syndrome means that even when the children are not nodding, many of them carry a ‘blemish’ and do not have the chance of ‘passing as normal’ in Goffman's sense. The reference to the children with derogatory terms, the stigma, and the separation of children due to fear of transmission are not easily disentangled – nonetheless, the provision of accessible treatment for nodding syndrome is likely to address (but not solve) several aspects of the problem because medication would reduce the frequency of nodding spells and seizures. This would in turn reduce salivating and other physical manifestations, which might reduce discrimination and stigma. Although stigma is not a rational response to illness, the physical manifestation of this syndrome makes the sufferer more visible to his surroundings. Medication also reduces the child's risk of getting burns, which has become an identifier of having nodding syndrome.

It has been documented by other studies that men in northern Uganda were left feeling disempowered by the protracted violent conflict and the loss of cattle and land that was their source of wealth (20, 23, 30, 31). Coupled with the financial responsibility of caring for many orphans after the war, this led to feelings of hopelessness and anger that were also found in this study, and a high rate of suicide.

The pain expressed in this study consists not only in their broken children, but also in their lost future. Acholi is an interdependent society and having no children means an end to one's lineage as there is no one to inherit land or to provide crucial support in old age (31). Parents who have several children affected with nodding syndrome are grieving more than their children's illness; it is a loss of pride and social value. The burden of care brings a reduction in economic activities, which affects entire families. It is vital to look into how parents cope with this loss of lineage and security and adjust to the socioeconomic changes brought by nodding syndrome. The findings of neglect in this study are consistent with reports that some parents had given up on their affected children (16).

As posttraumatic reactions take long to resolve, the effects of nodding syndrome may be seen long after new cases have stopped appearing. The suffering caused by nodding syndrome is collective and theories of causation are many, based in the understanding of war.

## Conclusion

The feelings of uncertainty and powerlessness are not caused by nodding syndrome alone; it is an accumulation of factors that left people wondering why a tragedy affected only their ethnic group yet again. Their explanations lie in the memory of war; this is how nodding syndrome is understood.

The years of lack of help for the very sick children reinforces the Acholi understanding that they are seen as of less value than other ethnic groups in Uganda. Interventions that help the affected children will help the community by reducing the collective suffering expressed throughout the themes.

## Footnotes

1 CWNS=child/children with nodding syndrome.

## References

[1] Sejvar JJ, Kakooza AM, Foltz JL, Makumbi I, Atai-Omoruto AD, Malimbo M (2013). Clinical, neurological, and electrophysiological features of nodding syndrome in Kitgum, Uganda: an observational case series. Lancet Neurol.

[2] Winkler AS, Friedrich K, Konig R, Meindl M, Helbok R, Unterberger I (2008). The head nodding syndrome – clinical classification and possible causes. Epilepsia.

[3] Iyengar PJ, Wamala J, Ratto J, Blanton C, Malimbo M, Lukwago L (2014). Prevalence of nodding syndrome – Uganda, 2012–2013. MMWR Morb Mortal Wkly Rep.

[4] Dowell SF, Sejvar JJ, Riek L, Vandemaele KA, Lamunu M, Kuesel AC (2013). Nodding syndrome. Emerg Infect Dis.

[5] Echwalu E Uganda: nodding horror – 12-year-old victim is tied to a tree for 13 hours every day.

[6] Idro R, Opoka RO, Aanyu HT, Kakooza-Mwesige A, Piloya-Were T, Namusoke H (2013). Nodding syndrome in Ugandan children – clinical features, brain imaging and complications: a case series. BMJ Open.

[7] Winkler AS, Friedrich K, Velicheti S, Dharsee J, Konig R, Nassri A (2013). MRI findings in people with epilepsy and nodding syndrome in an area endemic for onchocerciasis: an observational study. Afr Health Sci.

[8] Konig R, Nassri A, Meindl M, Matuja W, Kidunda AR, Siegmund V (2010). The role of Onchocerca volvulus in the development of epilepsy in a rural area of Tanzania. Parasitology.

[9] Spencer PS, Vandemaele K, Richer M, Palmer VS, Chungong S, Anker M (2013). Nodding syndrome in Mundri county, south Sudan: environmental, nutritional and infectious factors. Afr Health Sci.

[10] Tumwine JK, Vandemaele K, Chungong S, Richer M, Anker M, Ayana Y (2012). Clinical and epidemiologic characteristics of nodding syndrome in Mundri County, southern Sudan. Afr Health Sci.

[11] Winkler AS, Friedrich K, Meindl M, Kidunda A, Nassri A, Jilek-Aall L (2010). Clinical characteristics of people with head nodding in southern Tanzania. Trop Doct.

[12] Winkler AS, Wallner B, Friedrich K, Pfausler B, Unterberger I, Matuja W (2014). A longitudinal study on nodding syndrome – a new African epilepsy disorder. Epilepsia.

[13] Pion SD, Kaiser C, Boutros-Toni F, Cournil A, Taylor MM, Meredith SE (2009). Epilepsy in onchocerciasis endemic areas: systematic review and meta-analysis of population based surveys. PLoS Negl Trop Dis.

[14] Hotez PJ, Fenwick A, Savioli L, Molyneux DH (2009). Rescuing the bottom billion through control of neglected tropical diseases. Lancet.

[15] Foltz JL, Makumbi I, Sejvar JJ, Malimbo M, Ndyomugyenyi R, Atai-Omoruto AD (2013). An epidemiologic investigation of potential risk factors for nodding syndrome in Kitgum District, Uganda. PLoS One.

[16] Kitara D, Amone C (2012). Perception of the population in northern Uganda to nodding syndrome. J Med Med Sci.

[17] Mitchell KB, Kornfeld J, Adiama J, Mugenyi A, Schmutzhard E, Ovuga E (2013). Nodding syndrome in northern Uganda: overview and community perspectives. Epilepsy Behav.

[18] van Bemmel K, Derluyn I, Stroeken K (2014). Nodding syndrome or disease? On the conceptualization of an illness-in-the-making. Ethn Health.

[19] Baines EK (2007). The haunting of Alice: local approaches to justice and reconciliation in northern Uganda. Int J Transit Just.

[20] Roberts B, Odong VN, Browne J, Ocaka KF, Geissler W, Sondorp E (2009). An exploration of social determinants of health amongst internally displaced persons in northern Uganda. Confl Health.

[21] WHO U, WFP, UNFPA, IRC, Uganda MoH (2005). Health and mortality survey among internally displaced persons in Gulu, Kitgum and Pader districts, northern Uganda.

[22] Corbin JN (2008). Returning home: resettlement of formerly abducted children in northern Uganda. Disasters.

[23] Finnström S (2008). Living with bad surroundings.

[24] Kustenbauder M, Falola Toyin, Njoku Raphael Chijioke (2010). Northern Uganda: protracted conflict and structures of violence. War and peace in Africa.

[25] Namutebi J Uganda: Govt does not marginalize North – Minister.

[26] Virginia Braun VC (2006). Using thematic analysis in psychology. Qual Res Psychol.

[27] Ayebazibwe A Acholi sub-region desperate as children nod to their death.

[28] Bukuluki D-N K, Ovuga, Lien, Kaawa-Mafigiri (2012). Nodding syndrome in post conflict northern Uganda: a human security perspective. Glob Health Gov.

[29] Neuner F, Pfeiffer A, Schauer-Kaiser E, Odenwald M, Elbert T, Ertl V (2012). Haunted by ghosts: prevalence, predictors and outcomes of spirit possession experiences among former child soldiers and war-affected civilians in northern Uganda. Soc Sci Med.

[30] Dolan C (2009). Social torture: the case of northern Uganda, 1986–2006.

[31] Kizza D, Knizek BL, Kinyanda E, Hjelmeland H (2012). Men in despair: a qualitative psychological autopsy study of suicide in northern Uganda. Transcult Psychiatry.
